# The Hymen

**Published:** 1884-05

**Authors:** E. S. McKee

**Affiliations:** Adjunct lecturer on Gynæcology, Medical College of Ohio


### Article IV.

The Hymen. A Part of a Lecture by E. S. McKee, M.D., Adjunct lecturer on Gynæcology, Medical College of Ohio, March 29, 1884.

The hymen (from the Greek word meaning a pellicle) seu membrana virginitatis, called by the Germans Scheidenklappe, Jungfernhäutchen, Jungfernschatz, Jungfernschlœsslein, is a fold of the mucous membrane, situated at the outer orifice of the vagina in the normal condition of the sexual organs of the human female ; also present in the higher order of apes, in the ruminants and carnivora. Its shape is usually semilunar, circular, or parabolic.

Of varieties there are the following in the anatomical museum at Heidelberg :

I. The hymen semilunaris. The normal hymen.

II. The hymen circularis, with small central opening.

III. The hymen cribriformis, sieve-like, containing many holes like a watering pot.

IV. The hymen fibrinatus. Similar to the fringe-like appendages of the ostium abdominale of the tubæ fallopinæ. This form is the most important in a forensic view, as it may be taken for a normal hymen which has been torn.

V. The hymen imperforatus. This on account of the retensio mensium dependent on it, is a cause for surgical treatment. It may also prevent copulation.

Occasionally one finds the opening of the hymen divided into two parts by a perpendicular bridge from the concave border of the hymen to the meatus urinarius where it becomes fast. This sometimes appears as a species of bridle or small cord.

In the crescentic variety the concave border approaches more or less towards the urethra in such a way as to contract the vagina behind, and then it almost always gives way in coition.

In the circular variety the free border is much thinner than the other, often being fringed, and leaving an opening which is sometimes round and sometimes slightly elongated, and in general situated somewhat nearer the anterior than the posterior wall of the vagina. In some cases there exists an upper anterior and a lower posterior opening with simply a band lying transversely across the vagina. We have also in rare cases a second hymen existing above the first.

The hyman imperforatus which is generally accompanied by a thickening of the membrane is called atresia vaginæ membranacea seu externa. It is proper to differentiate between atresia interna or completa, which depends either on an incomplete development of the vagina from its entire absence or from a diseased condition of the same in consequence of ulcerative processes.

It has been asserted and disputed that the hymen in the negress is not in the same position that it is in the white woman, that is, that it is situated from one-half to two inches up the vagina, instead of over the orifice. Dr. Turnipseed, of South Carolina, claims that the hymen in the negro race is not at the entrance of the vagina, as in the white woman, but as above stated. He reports the examination of eleven cases from 9 to 12 years old, where the hymen is 1½ to 2 inches up. He claimed this to be "one of the anatomical relations Providence has given us to show the non-unity of the races."

Dr. Hyatt, of North Carolina, denies the assertion of Dr. Turnipseed. He has examined 1,000 negresses and has found no marked differences between them and the whites, with the exception of the greater size of the labiæ minoræ of the negress.

Dr. Fort, of Tennessee, reports 6 cases in which he claims to have found the hymen 1 to 2 inches from the vulva. In several cases the hymen was very dense.

Congenital absence of the hymen occurs sometimes as an anomaly. Hyatt reports two such cases. Forensically, this fact is well to know.

Casper says that in children there is often a hymen shaped like a keg or jug, generally 1 to 1½ lines broad. This formation during childhood often remains, in the cases of idiot girls, throughout life; also a cuff-like hymen is found in this same class. This appears to be more fleshy than membranous.

Sometime there are found on each side lips, numbering four or five, placed like the tiles on a roof. These are endowed with varied powers of resistance. The openings were found to be large enough to admit of a finger. They were found not always to be round or oval, but bridged over with a band or lattice work. He adds another form in which there are numerous lips overlapping each other with a heart-shaped opening In other cases of lip formation he notices what appear to be reduplications of the labiæ minoræ.

These anomalies of the hymen do not have any effect on the other parts of the female genitalia, they remaining perfect in every particular.

As to the hymen in embryo, Scanzoni says: "The hymen, as is known, is completely absent. In the new born child it forms but a minute fold of the mucous membrane, which is insensibly elevated until the age of puberty." This latter fold of the mucous membrane simply marks the place where the hy..en will be when the sexual organs obtain development.

The hymen does not appear, according to Dr. Dolm, until the nineteenth week, not being visible at the sixteenth week. It does not develop on the upper border of the urogenital sinus, but the lowest segment of the urogenital tube. At the middle of the embryonic period, the inner surface of the urogenital tuoe shows considerable excess of tissue. This shows itself in the curve of the vagina, elongation of the posterior wall, the growth of the papillæ and rugæ. Further on, this hyperplasia extends to the tissue of the external covering, and leads to the formation of an apron-like fold, which gradually grows in breadth. In accordance with its greater proliferation, the posterior vaginal wall plays a prominent part in the formation of this fold.

Von Hoffman, of Wiesbaden, formulated his conclusions of observations on embryos as follows:

I. The hymen should not be considered as a growth springing independently from the wall of the genital tube, but is an accessory product to the formation of the vagina, the lower portion of which touches the lower or dorsal surface of the allantois, gradually verges into the urogenital sinus, and then forms the floor of the vestibule which is chiefly represented by the hymen.

II. Every hymen, in his opinion, originally possessed a double perforation, and probably the two openings are the former outlets of the Wolffian ducts in the urogenital sinus.

The hymen is attached to the inferior and lateral borders of the orificium vaginæ, and is of a semilunar shape, with its convex border below or posteriorly, and looking toward the perineum, and its concave border above or anteriorly, looking toward the urethra. It is constituted of a mucous fold, containing between its lamellæ a layer of cellular tissue enclosing numerous elastic fibers, and some muscular bundles of organic life. Some blood-vessels ramify in it. The epithelium is laminated, tesselated, and nearly the same thickness as that of the vestibule, 0.3–0.5 m. The delicate but highly vascular and nervous mucous membrane bristles with close set, conical, divided and undivided papillæ, that project into the epithelium, and are 0.2–0.3 m. long.

In an examination of the hymen, to see it plainly, the thighs must be abducted, and the labiæ minoræ must be pulled downward as well as apart, thus pulling the frenulum labiorum out of the way. In moderate abduction, the shape is that of a half moon. If the abduction is increased, this shape is destroyed. Rupture of the hymen occurs, in the vast majority of the cases, on the first approach of the male (defloratio). There are hymens, however, which the male organ is unable to rupture. Intercourse in these cases must remain vulvar, unless there is surgical interference.

Cases are on record where this vulvar intercourse was carried on for years, until the female finally became pregnant and came to term. The accoucheur's attention was called to the existing circumstances either through his inability to make an examination, or when through the inability of the woman to expel her child, the knife was called into use. In other cases, that part of the vaginal entrance not covered with the hymen being large and the hymen probably tough with the male organ small and weak, connection may be completed, and the hymen remain intact. Baudelocque saw two, and Blundell four cases where the patient became pregnant and arrived at term with the hymen unruptured. A specimen is on exhibition in the museum at Halle, where the mother had given birth to a seven months' fœtus, and the hymen remained perfect.

On the contrary, the rupture of the hymen may occur in various other ways than through sexual intercourse—onanism; a fall with the feet widely separated; riding horseback after the manner of men; ulcerative diseases; flooding; the passage of a tumor from within, or the examining finger from without. Hyrtle reports a case where a young girl was sawing wood. She was thrown upon the saw by a passing wagon, and the handle driven into the vagina, rupturing the hymen. *Apropos*, last summer I was sitting in my front office by the half-open door, reading, when quickly an honest, anxious, unsophisticated seeker after truth, evidently just married, stepped in and was seated beside me before I had time to look up. "Doctor," said he, "can a woman lose her virgin any other way than by being with a man?" I replied: "Yes, she may lose it by flooding, the passage of an instrument, the finger, or violent or sudden exercise." He continued: "Ought a woman, if she is all right, 21 years old, of full size, to complain of pain and it hurting her when a man goes to her the first time?" I replied, "She ought, and in some cases this pain lasts for some time; in one case, I gave chloroform to a newly married woman whose husband had been unable to have satisfactory connection during the two weeks they had been married, every attempt giving the wife the most exquisite pain. The connection was perfected, and when recovering from the chloroform, yet semi-unconscious, she begged him to repeat the act which before she had resisted with terror. I repeated the chloroform in a few days, subsequent to which all went smoothly." The young man said: "I just wanted to know," handed me a fee, bade me good-day, and left as suddenly as he came.

Concerning the function of the hymen, it seems to have been relegated as a *signum anatomicum* of innocence and virginity, as such, however, it has in recent days lost much of its credit. Hyrtle gets off the following "Irishism": Who has it not, can never obtain it. Yet in all times, in all ages, and among all races, civilized and uncivilized, the hymen has been held as a sign of the virtue and morality of the holder. The old Jews proudly carried the shirt of the newly married about among the relatives and friends, showing the traces of blood. Such a custom is still in vogue in Naples, as the writer found on questioning physicians when there. The Ehrenhemde of the Germans, the camiscci del onore of the Italians, the shirt of honor. The Mohammedans held the flow of blood as a criterion of previous virtue when the flow occurred in *fremis nuptiis.*

The ancient Egyptians cut the hymen through to have it out of the way. Athanasius says that the Phœnicians turned the bride over to a favorite slave to be relieved of her virginity. Under the Emperor Tibernius a law was promulgated that no virgin should be hanged; she must first be relieved of her hymen by the hangman. On the contrary, the renowned courtesan Marion de Lorme, who died in consequence of the getting rid of her seventh fatherless child, was buried with the virgin's crown. The pastor of St. Sulpice, however, after the funeral had the politeness to tear it down and tramp it under foot.

A Roman satyr who wrote of the sexual sins of his age (probably his own) had a little maiden say: "*Junonum meam ratam habeam, si unquam mesmerum me virginem fuissi.*" The hymen being a hindrance to sexual intercourse, the weak-backed and demoralized people of ancient times had the rupture made by the priests or little ivory idols.

Shakespeare has to say in reference to the hymen: "The longer kept the less worth." Among the ancient Greeks the long possession of the hymen meant its possessor to be extremely ugly. They named the old maids furies. The following were the words of the holy Hieronymus: "*Difficiles res virginates idolque rara.*"

In some Asiatic tribes a widow is preferred to a virgin by the would-be husband. *Virgo intacta* has no charms for him. He simply looks at the matter very practically. The widow, he thinks, has no mechanical hindrance to coition, and knows more of wifely and household duties. A man who has a supply of widows as daughters can realize twice as largely on them for their second marriage as he did for their first. One of their number handed his name down to history by being so benevolent as to give his widowed daughter to his friend for a wife at the same price a virgin would bring.

The following in regard to the hymen, or virginity, is gleaned from the Scriptures:

Speaking of the marriage of the higher priests, Lev. xxi, 13: And he shall take a wife in his virginity.

14. A widow, or a divorced woman, or profane, or an harlot these shall he not take, but he shall take a virgin of his own people to wife.

And further, Deut. xxii. 13: If a man take a wife and go in unto her and hate her.

And give occasion of speech against her and bring up an evil name upon her, and say: I took this woman, and when I came unto her I found her not a maid:

15. Then shall the father of the damsel and her mother take and bring forth the tokens of the damsel's virginity unto the elders of the city in the gates.

16. And the damsel's father shall say unto the elders: I gave my daughter to this man to wife and he hateth her.

17. And lo, he hath given occasion of speech against her, saying, I found not thy daughter a maid:

18. And yet here are the tokens of my daughter's virginity, and they shall spread the cloth before the elders of the city.

19. And the elders of the city shall take that man and chastise him.

20. And they shall amerce him in an hundred shekels of silver, and give them unto the father of the damsel, because he hath brought up an evil name upon a virgin of Israel; and she shall be his wife, he may not put her away all his days.

21. But if this thing be true, and the tokens of virginity be not found for the damsel:

22. Then shall they bring out the damsel to the door of her father's house, and the men of her city shall stone her with stones that she die.

And still further, Deut. xxii, 23: If a damsel that is a virgin be betrothed unto an husband, and a man find her in a city and lie with her:

24. Then ye shall bring them both out unto the gate of that city, and ye shall stone them with stones that they die; the damsel that she cried not, being in the city ; and the man because he hath humbled his neighbors wife ; so thou shalt put away evil from among you ; but if a man find a betrothed damsel in a field, and the man force her and lie with her, then the man only that lay with her shall die.

26. But unto the damsel thou shalt do nothing ; there is in the damsel no sin worthy of death ; for as when a man riseth against his neighbor and slayeth him, so is this matter.

27. For he found her in the field, and the betrothed damsel cried, and there was none to save her.

28. If a man find a damsel that is a virgin, which is not betrothed, and lay hold on her, and lie with her, and they be found,

29. Then the man that lay with her shall give unto the damsel's father 50 shekels of silver, and she shall be his wife ; because he hath humbled her, he may not put her away all his days.

In all times the taking of the hymen unlawfully has been visited with severe punishment. Among the Jews, if the girl was engaged ; among the Athenians, Romans, and old French, and English, and in many of the United States in their earlier days, the offense was punishable with death. In New York, by the law of 1787, rape of a child under 10 years of age was punishable with death ; in 1810 it was changed to imprisonment for life. Also in Illinois and Massachusetts the punishment formerly was death.

Among the old Welsh, he who robbed a maiden of her hymen, there being two witness to the same, was required to present to his sovereign a piece of silver as high as the sovereign's mouth and as thick as his little finger. In the Isle of Man, in ye olden times, there existed a very wise custom. The criminal was brought into a public place, and the girl whom he had disgraced was given a sword, a whip, and a ring. His punishment lay entirely in her hands—she could either kill, whip, or marry him.

The diagnosis of rupture of the hymen in its medico-legal aspect, whether the rupture be moral or mechanical, or, as the French have it, *virginite ou pucellage*, allows for a great display of tact on the part of the examining physician. This diagnosis between the moral and mechanical rupture of the hymen is so difficult as to be almost impossible.

Prolapsus of the vagina may make it appear that there is no hymen present. The tendency of modern writers is, I think, to lay too little stress on the presence or absence of the hymen.

There are, of course, many ways in which the hymen may be removed other than moral, but their occurrence is rare. They are very rare. One would think from the opinions expressed by most writers, that they had been exceedingly unfortunate, and had not found a hymen, therefore they concluded that all women were unvirtuous. Like the young man who has spent his substance with harlots, he thinks all women are harlots, or, to cover his own sins, pretends that virtue is unknown except in newly born babes. In some localities, and among some people, this may be true, but among others it is not. The writer remembers distinctly how a clinical teacher in Vienna called a class of practitioners, and with great pride, showed the presence of a hymen in a female 24 years of age.

It was dilated on largely, the teacher evidently believing that his hearers had scarcely, if ever before seen such a membrane, and fearful that they, possibly himself, would never again see the like.

Devergie says: "When not found, in 999 cases out of 1,000, defloration has taken place. He regards the restoration of the hymen as one of the many fables."

Casper seems to know whereof he speaks when he says: "I must declare that when a forensic physician finds a hymen still preserved, even its edges not being torn, and along with it (in young persons) a virgin condition of the breasts and external genitals, he is justified in giving a positive opinion as to the existence of virginity, and *vice versa*."

Fresh rosy lips, bright, beaming eyes, with a free yet modest look, the poetical sign of virginity, has, with scientists, not much credit. Still less has the old Roman sign, the swelling of the neck after matrimony. This made it a part of the marriage ceremony to measure the neck with a thread before and after the first dissipations. They thought if the bride's neck swelled after marriage, she had not been accustomed to sexual pleasures. Did it not, the inference was that she had.

The remnants of the hymen after it is broken through are drawn into the three little thick, fleshy warts. Immediately after the first coition, they may be seen as irregular bloody lips, and after a week or so contract themselves into the carunculæ myrtiformes (*quod figuram habeant Baccarum myrti*). In general there are but three, one on the back wall of the vagina, the other two, one on either side. They sometimes resemble so much the comb of a cock that they simulate not a little condylomata.

They may become inflamed and hypertrophied, and become a hindrance to parturition and require removal by the knife.

Barnes, Robert—Diseases of Women. London, pp. 59, 64, 176, 105, 181, 189, 190.

Brown, Chas. W.—*Phila. Med. Times*, Nov. 8, 1873; also supplement *Obstetrical Journal of Great Britain and Ireland*, p. 12.

Casper, Johann Ludwig—Handbuch d. Gerichtliche Medezin. Berlin, 1883, vol. i; also translation by Sydenham Society.

Cazeaux, P.—Midwifery, p. 59, 586.

Beck—Medical Jurisprudence, 1823, pp. 73, 74; also Elements of Jurisprudence, London, 1825, p. 65.

Deuteronomy—xxii, 15, 17, 20, 25.

Gray, Henry—Anatomy, 1883, p. 900.

Hart & Barbour—Manual of Gynæcology, 1883, pp.4, 6, 480 483, 488, 489.

Hyrtle—Anatomie, p. 729; also Topographischer Anatomie.

Hoffmann E.—*Vierteljahrcschrift für Gerichtlichen Medizin*, N. F., Bd. 12, S. 229.

Henkes—*Zeitschrift der Staatsartznei*, 1843, ii, 149–44, i, 259, *N. Y. Med Gaz.*, June, 1858, pp. 217, 220.

Heitzman C.—Anatomie, iv, p. 78, 1875.

Judges—xi, 37, 38.

Leviticus—xxi, 13.

Mayer—Neapel und die Neapolitaner, i, Oldenburgh, 1840, S. 319.

Osiander, F. B.—Denkwürdigkeiten, Gottingen, 1875.

Paschlæ—Hymen Columnatus et Vagina Duplex, *Wiener Med. Presse*, 1877, No. i.

Playfair, W. S.—Midwifery, 1880, pp. 43, 44.

Quain—Anatomy, 1882.

Skrziczka—Die Form des Hymen bei Kindern, *Vierteljahreschrift für Gerichtlichen u. Œffentlichen Med.*, ii, 47, 1866.

Stricker, S.—Manual of Histology, new Sydenham Society, 972, vol ii, p. 321.

57 W. 7th Street, Cincinnati.

---

We call attention to the following circular letter:

Indianapolis, April 10, 1884.

Messrs. Editors:—The next meeting of the Mississippi Valley Medical Association (formerly the Tri-State Medical Society), will be held at Springfield, Illinois, in September, 1884.

Wishing to make the work of the society more interesting and valuable, if possible, than ever, we desire to have papers from the more prominent members of the profession in this portion of our country. We therefore hope you may favor us with a paper. If your time is too much occupied to write a paper, we sincerely hope you will favor us with your presence at the meeting and your counsel in the discussions. As this is not a delegated society, every reputable member of the profession in the Mississippi Valley is eligible to membership by attending the meetings. We hope you will induce your friends to attend and assist in advancing the science of medicine.

Should you be pleased to contribute a paper, you will please communicate the title thereof to Dr. Chas. D. Pearson, Chairman of Committee, 30 East Ohio St., Indianapolis, Ind.

Very truly yours, etc.,

Chas. D. Pearson, m.d., Indianapolis, Ind.,
Wm. A. Byrd, m.d., Quincy, Ill.,
A. M. Owen, m.d., Evansville, Ind.,
Committee on Programme.

