# Errata

**Published:** 1884-06

**Authors:** 


### ERRATA.

In the article on the Hymen, by Dr. E. S. McKee, in the May number of the MEDICAL JOURNAL AND EXAMINER, the following corrections should be made: For camisca, read camiscci; for primis, read fremis; for juonem, read juonum; for memiserem, read mesmerum; for virginitas, read virginates. In enumerating the punishments for rape of various States and nations, the fact that death is the punishment in Texas was omitted.

